# Do actual tidal volumes differ from prescribed tidal volumes?

**DOI:** 10.1186/cc10699

**Published:** 2012-03-20

**Authors:** R Kleijn, B Kalkman, N Verburg, H Oudijk, B Van Vondelen, M Luttmer, I Slagers, M Ruijters, I Meynaar

**Affiliations:** 1 Reinier de Graaf Groep, Delft, the Netherlands

## Introduction

Studies have shown that the selection of incorrect tidal volume can cause ventilator-induced lung injury and increased mortality [1]. This study was done to determine if the actual tidal volume (aVt) differs from the prescribed tidal volume (pVt) based on predicted body weight (PBW).

## Methods

The ICU is a 10-bed intensivist-led unit in a 500-bed teaching hospital. All consecutive patients receiving invasive mechanical ventilation in June 2011 were included. Patients with noninvasive ventilation or with continuous positive airway pressure only were excluded. The ICU has a mechanical ventilation protocol that prescribes tidal volume to be between 6 and 8 ml/kg PBW. A table with prescribed tidal volumes based on PBW is available at the bedside throughout the ICU. All patients were ventilated with Drager Evita XL ventilators on pressure support (ASB) or pressure control mode (BIPAP). During the study period we compared the aVt with the pVt each day at 0, 6, 10, 14, 18 and 22 hours for all patients.

## Results

Seventeen patients with mean age of 70.2 years (SD 14.1) and median APACHE IV expected mortality of 31% (IQR 14 to 70), 10 admitted for medical reasons and seven for surgical reasons, and ventilated for 4 days median (IQR 3 to 6) fulfilled inclusion criteria and were included in the study. Results of tidal volume measurements are shown in Table 1.

**Table 1:** Tidal volume measurements

Total number of aVt measurements	286
Number of aVt measurements per patient (IQR)	12 (4 to 20)
aVt <6 ml/kg PBW	25 (9%)
aVt 6 to 8 ml/kg PBW	156 (58%)
aVt 8 to 10 ml/kg PBW	82 (29%)
aVt >10 ml/kg PBW	23 (8%)
Mean aVt per kg PBW (SD)	7.85 (1.23)

aVt, actual tidal volume; PBW, predicted body weight.

## Conclusion

In this small single-centre study, the mean aVt is between 6 and 8 ml/kg PBW as prescribed, but only 58% of measured tidal volumes are indeed between 6 and 8 ml/kg PBW.
